# Supplementary figures and images for: Schizosaccharomyces pombe Rtf2 is important for replication fork barrier activity of RTS1 via splicing of Rtf1
Source: eLife. 2023 Aug 24;12:e78554. doi: 10.7554/eLife.78554 (PMC10473836; doi:10.7554/eLife.78554)

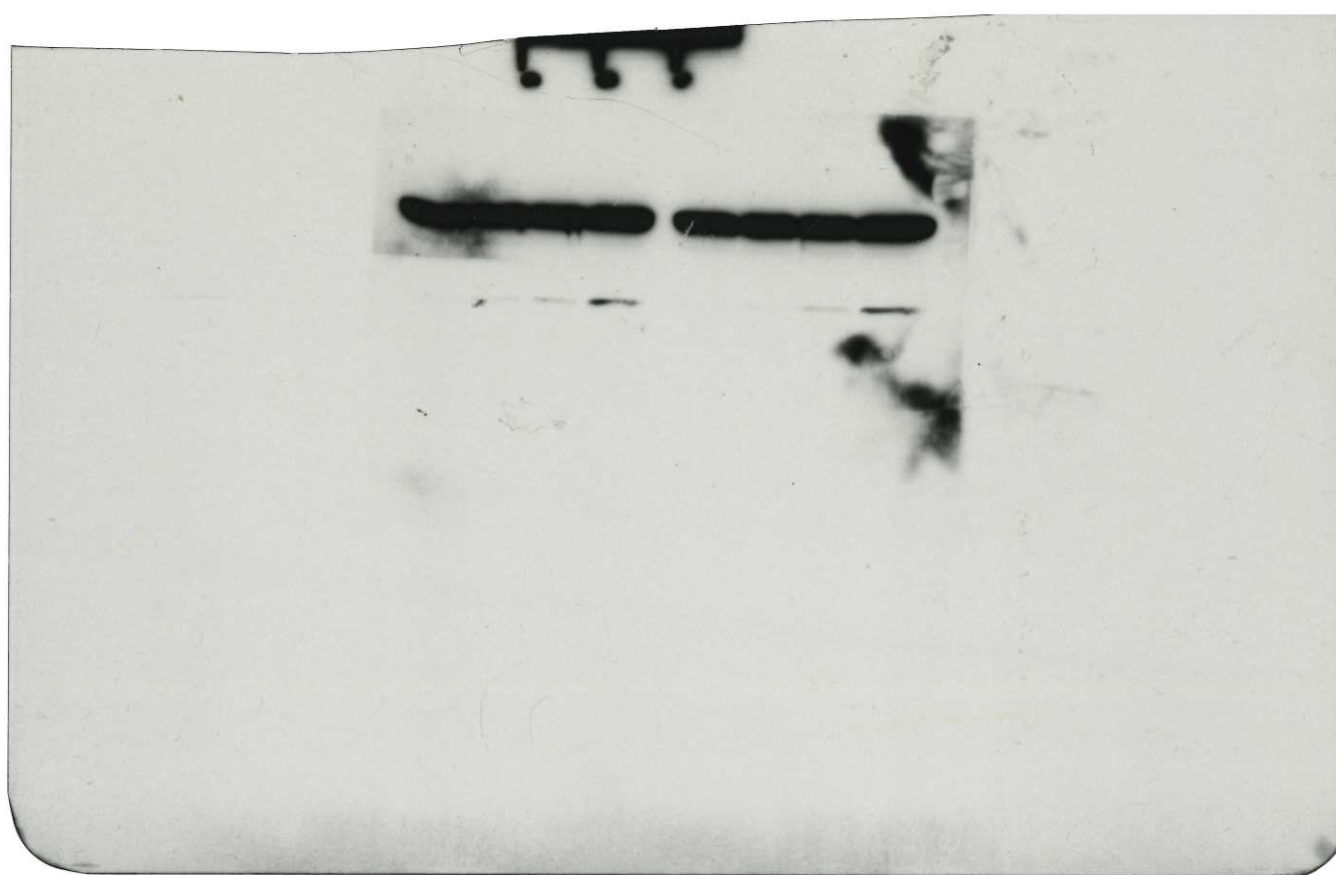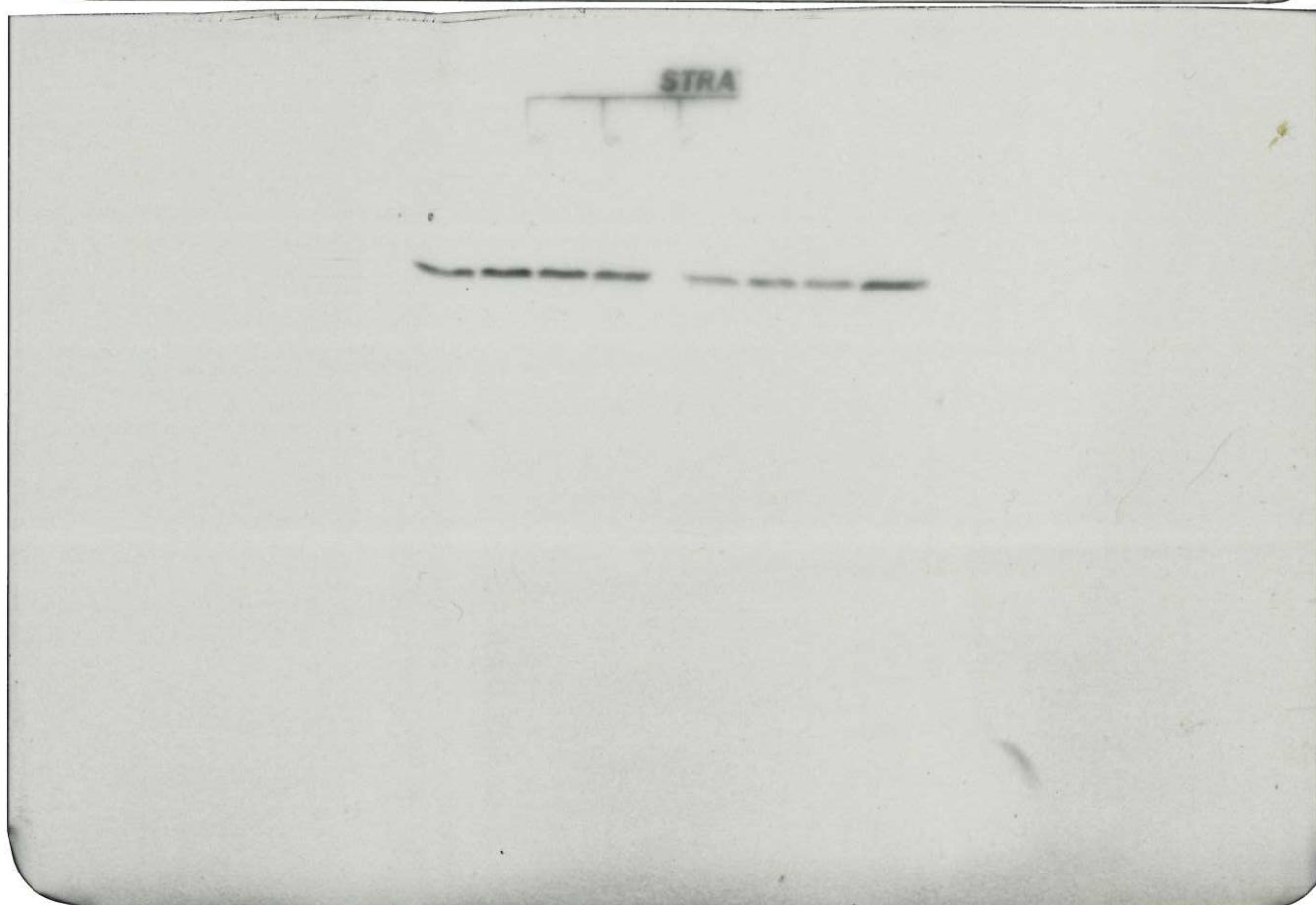

Supplement: Figure 2—figure supplement 1—source data 1. [file elife-78554-fig2-figsupp1-data1.zip › Fig2-fig-sup-1-source/Figure 2-figure supplement 1-source data 1.pdf]

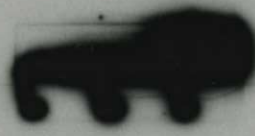

VS-141, 142-5A  
 VS-141-5A  
 WT

- 170
- 130
- 100
- 70
- 55
- 45
- 35
- 25

2-VS

8/11/21

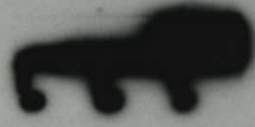

VS-141, 142-5A  
 VS-141-5A  
 WT

- 170
- 130
- 100
- 70
- 55
- 45
- 35
- 25

2-VS

8/11/21

Supplement: Figure 3—source data 1. [file elife-78554-fig3-data1.zip › Fig3-source/Figure 3-source data 2.pdf]

1  
 2  
 3  
 4  
 5  
 6  
 7  
 8  
 9  
 10  
 11  
 12  
 13  
 14  
 15  
 16  
 17  
 18  
 19  
 20  
 21  
 22  
 23  
 24  
 25  
 26  
 27  
 28  
 29  
 30  
 31  
 32  
 33  
 34  
 35  
 36  
 37  
 38  
 39  
 40  
 41  
 42  
 43  
 44  
 45  
 46  
 47  
 48  
 49  
 50  
 51  
 52  
 53  
 54  
 55  
 56  
 57  
 58  
 59  
 60  
 61  
 62  
 63  
 64  
 65  
 66  
 67  
 68  
 69  
 70  
 71  
 72  
 73  
 74  
 75  
 76  
 77  
 78  
 79  
 80  
 81  
 82  
 83  
 84  
 85  
 86  
 87  
 88  
 89  
 90  
 91  
 92  
 93  
 94  
 95  
 96  
 97  
 98  
 99  
 100

0.1 -  
 0.5 -  
 1.0 -  
 2.0 -  
 5.0 -  
 10.0 -  
 20.0 -  
 50.0 -  
 100.0 -  
 200.0 -  
 500.0 -  
 1000.0 -

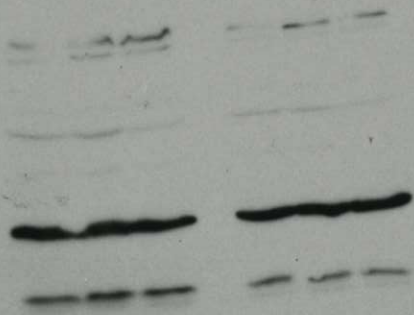

5060-20

15/11/90

Supplement: Figure 3—source data 1. [file elife-78554-fig3-data1.zip › Fig3-source/Figure 3-source data 3.pdf]

Figure S5 - tubulin

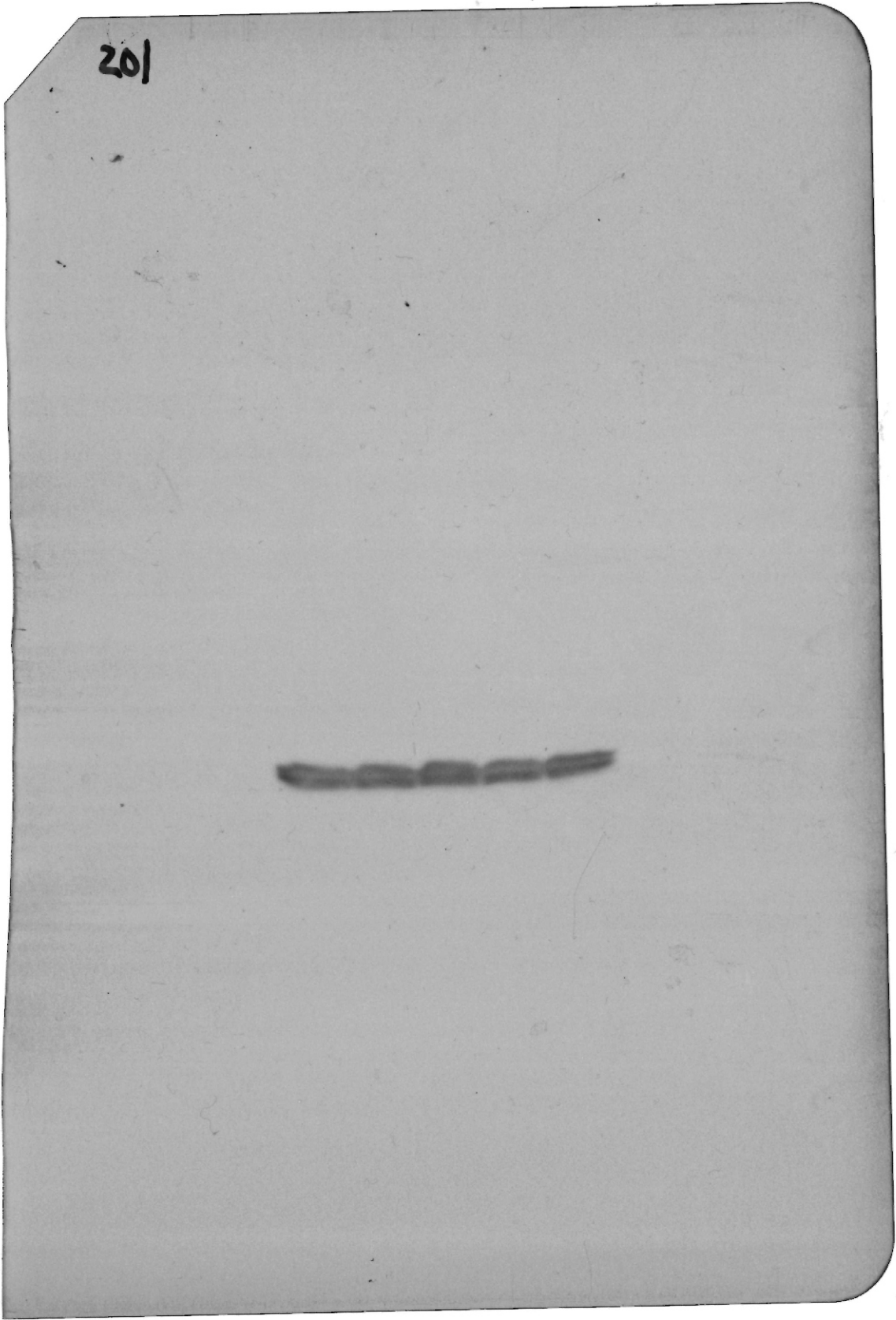

Supplement: Figure 4—figure supplement 2—source data 1. [file elife-78554-fig4-figsupp2-data1.zip › Figure 4 fig-sup-2 Source data/Figure S5 - tubulin[29].pdf]

Figure S5 - V5

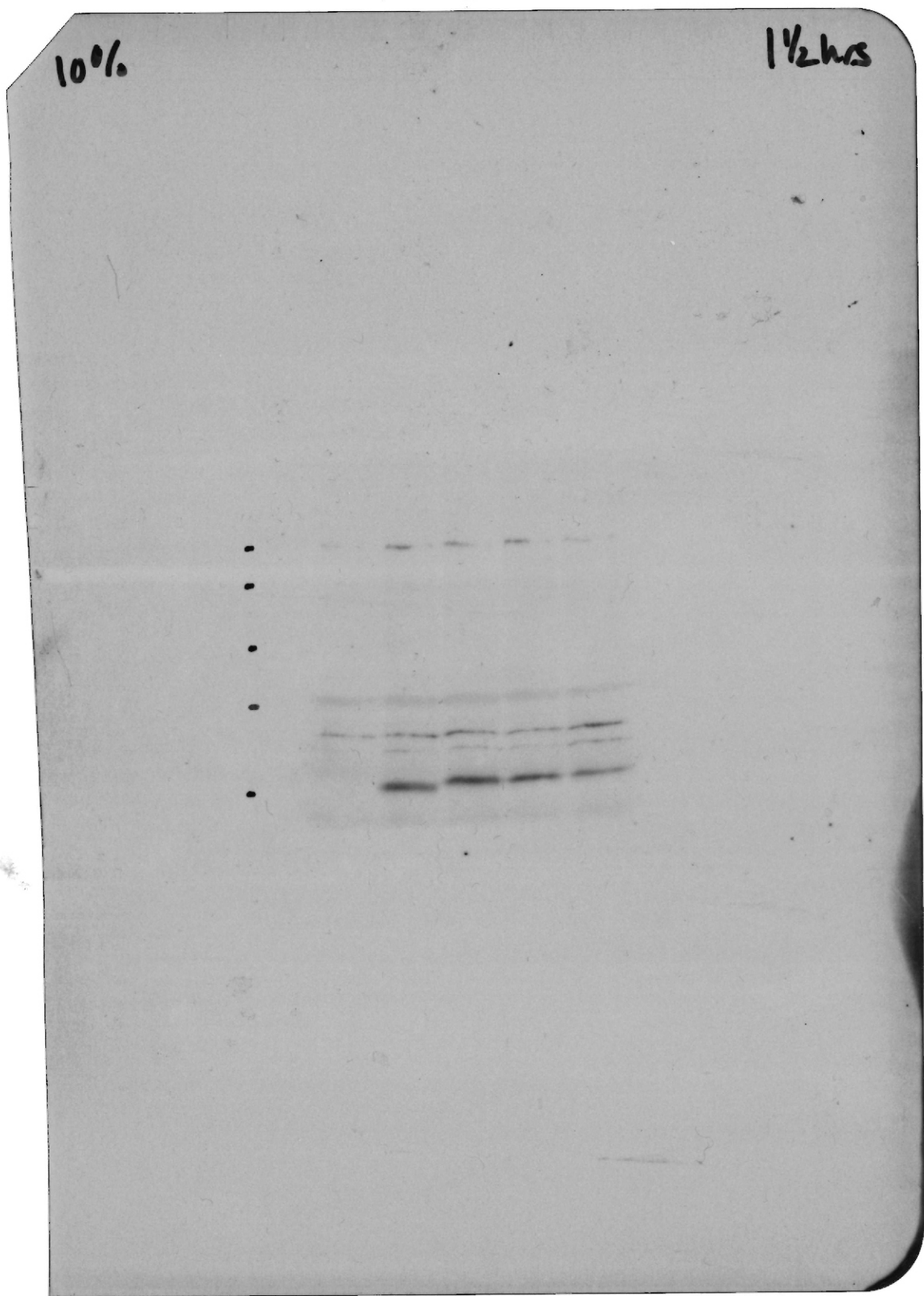

Supplement: Figure 4—figure supplement 2—source data 1. [file elife-78554-fig4-figsupp2-data1.zip › Figure 4 fig-sup-2 Source data/Figure S5 - V5[86].pdf]
