# Supplementary material for: Schizosaccharomyces pombe Rtf2 is important for replication fork barrier activity of RTS1 via splicing of Rtf1
Source: eLife. 2023 Aug 24;12:e78554. doi: 10.7554/eLife.78554 (PMC10473836; doi:10.7554/eLife.78554)

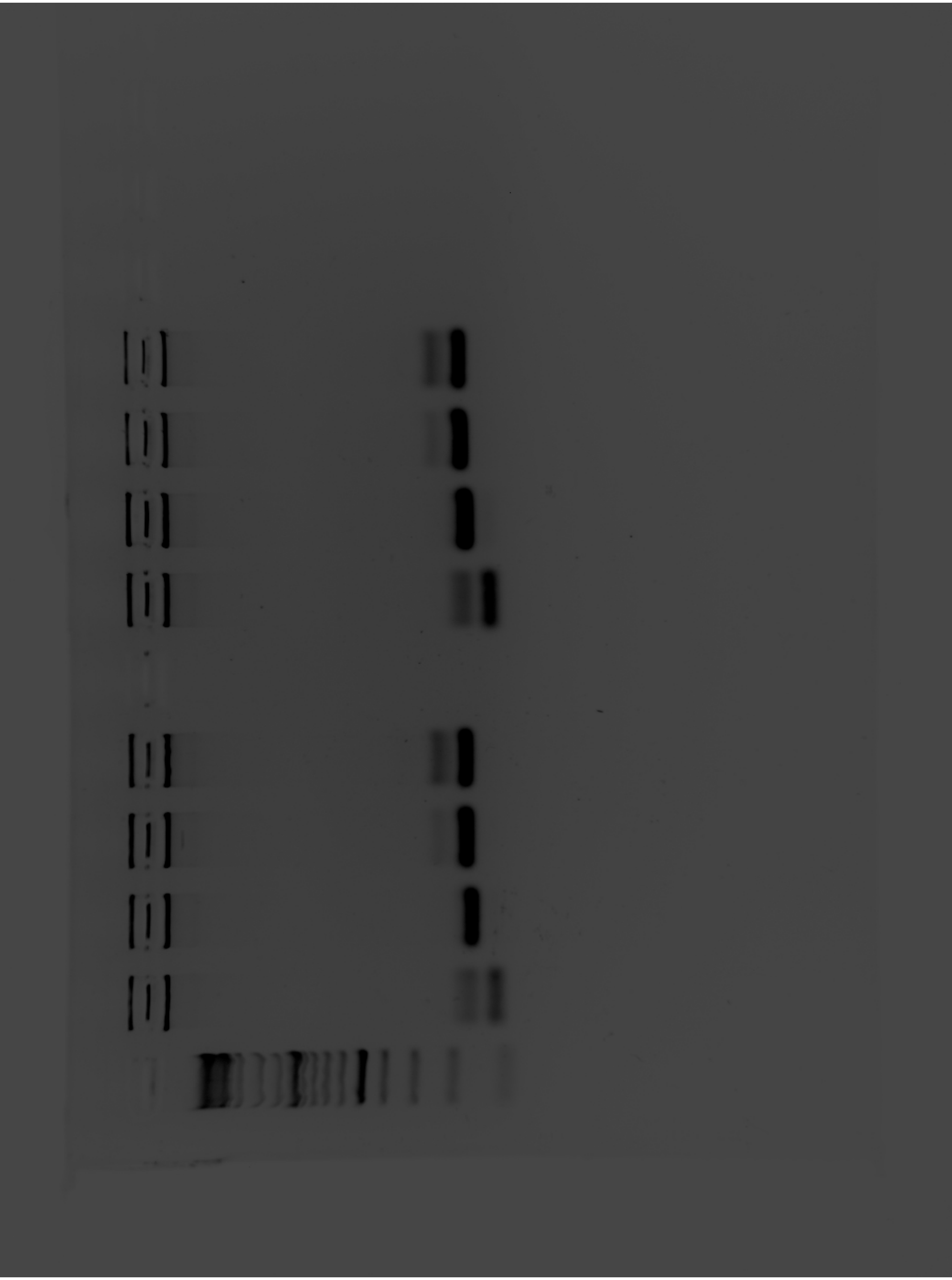

Supplement: Figure 3—source data 1. [file elife-78554-fig3-data1.zip › Fig3-source/Figure 3-source data 1.pdf]
